# Mut-Map: Comprehensive Computational Pipeline for Structural Mapping and Analysis of Cancer-Associated Mutations

**DOI:** 10.1093/bib/bbae514

**Published:** 2024-10-16

**Authors:** Ali F Alsulami

**Affiliations:** Department of Biochemistry, Faculty of Science, King Abdulaziz University, Jeddah, Saudi Arabia

**Keywords:** somatic mutations, mutation mapping, mutation impact analysis, structural bioinformatics, cancer genomics, computational pipeline

## Abstract

Understanding the functional impact of genetic mutations on protein structures is essential for advancing cancer research and developing targeted therapies. The main challenge lies in accurately mapping these mutations to protein structures and analysing their effects on protein function. To address this, Mut-Map (https://genemutation.org/) is a comprehensive computational pipeline designed to integrate mutation data from the Catalogue Of Somatic Mutations In Cancer database with protein structural data from the Protein Data Bank and AlphaFold models. The pipeline begins by taking a UniProt ID and proceeds through mapping corresponding Protein Data Bank structures, renumbering residues, and assessing disorder percentages. It then overlays mutation data, categorizes mutations based on structural context, and visualizes them using advanced tools like MolStar. This approach allows for a detailed analysis of how mutations may disrupt protein function by affecting key regions such as DNA interfaces, ligand-binding sites, and dimer interactions. To validate the pipeline, a case study on the TP53 gene, a critical tumour suppressor often mutated in cancers, was conducted. The analysis highlighted the most frequent mutations occurring at the DNA-binding interface, providing insights into their potential role in cancer progression. Mut-Map offers a powerful resource for elucidating the structural implications of cancer-associated mutations, paving the way for more targeted therapeutic strategies and advancing our understanding of protein structure–function relationships.

## Introduction

Understanding the implications of genetic mutations is pivotal for advancing our knowledge of disease mechanisms, drug resistance, and therapeutic interventions [1]. Mutations, which are alterations in the nucleotide sequence of a genome, can lead to changes in the amino acid sequence of proteins, potentially altering their structure and function [2]. These alterations can have profound effects on cellular processes and can be a driving force behind various diseases, including cancer, neurodegenerative disorders, and genetic syndromes [3].

One of the primary challenges in studying mutations is not merely identifying their presence but determining their structural and functional consequences [4]. The location of a mutation within the 3D structure of a protein can provide critical insights into its potential impact. For instance, mutations occurring in the core region of a protein might disrupt its folding and stability, while those at the protein–protein interface could affect oligomerization or interactions with other biomolecules [5]. Similarly, mutations in the active site or ligand-binding regions can directly influence enzymatic activity or binding affinity, respectively [6].

Given the intricate relationship between a protein’s structure and function, mapping mutations onto protein structures is an essential step in understanding their biological significance. The Protein Data Bank (PDB) serves as a crucial repository, offering a wealth of structural data for proteins, which can be leveraged to visualize and analyse mutation sites [7]. The Catalogue Of Somatic Mutations In Cancer (COSMIC) is a comprehensive and authoritative resource dedicated to documenting somatic mutations in human cancer [8]. COSMIC 3D serves as a critical source of mutation data, enabling the integration of detailed mutational landscapes with protein structural information [9]. However, the process of integrating mutation data with structural information is complex and requires sophisticated computational tools and pipelines.

The ability to analyse and predict the impact of mutations is increasingly important in the era of precision medicine. Personalized treatments based on an individual’s genetic makeup require detailed knowledge of how specific mutations influence disease mechanisms and drug responses [10]. For example, mutations in the BRCA1 and BRCA2 genes are associated with a high risk of breast and ovarian cancers [11, 12], and understanding these mutations has led to the development of targeted therapies such as PARP inhibitors [13]. Pino *et al*. [14] explored the structural context of millions of human cancer–associated missense mutations, revealing distinct mutational signatures between tumour suppressors and oncogenes. They found that tumour suppressors are enriched in structurally damaging mutations, reflecting loss-of-function mechanisms, while oncogenes harbour structurally mild mutations consistent with gain of function. Despite the challenge of distinguishing oncogenes from noncancer genes using structural damage alone, the study demonstrated that clustering mutations in 3D space, particularly when recurrence is considered, effectively predicts oncogenes, offering insights for identifying novel candidate driver genes in cancer research. Thus, a deep understanding of mutations and their structural implications is critical for developing effective, personalized treatment strategies.

Map-Mut pipeline automates the entire workflow, from UniProt ID input to mutation mapping across all relevant PDB structures and AlphaFold. This gene-level approach ensures that all structural contexts are considered, providing a more extensive and accurate mapping of mutations. The pipeline offers a robust framework for systematically gathering and classifying data on the locations of mutations within protein structures, which is crucial for machine learning applications.

The pipeline generates detailed and accurate datasets that capture the spatial context of each mutation. These datasets are invaluable for training machine learning models to predict mutation functional impacts. For instance, models can be developed to distinguish between stabilizing and destabilizing mutations based on their structural context or to predict the likelihood of a mutation disrupting protein–protein interactions, protein–DNA interactions, or protein–ligand interactions.

Mut-Map integrates mutation mapping on the experimental and modelled structures, uniquely mapping mutation locations around ligands, between dimers, at protein–DNA interfaces, and within core regions, giving detailed mutation classifications and structural context for large datasets. It also builds mutant structures and ensures accurate residue renumbering, covering both experimental and AlphaFold models. In contrast, tools such as COSMIC-3D only render mutations on available PDB structures without modelling or precise renumbering, leading to potential inaccuracies. In this paper, I describe the development and validation of the Map-Mut pipeline, demonstrating its utility in facilitating mutation analysis and advancing our comprehension of protein structure–function relationships.

## Methods

### Pipeline development

**Figure 1:**
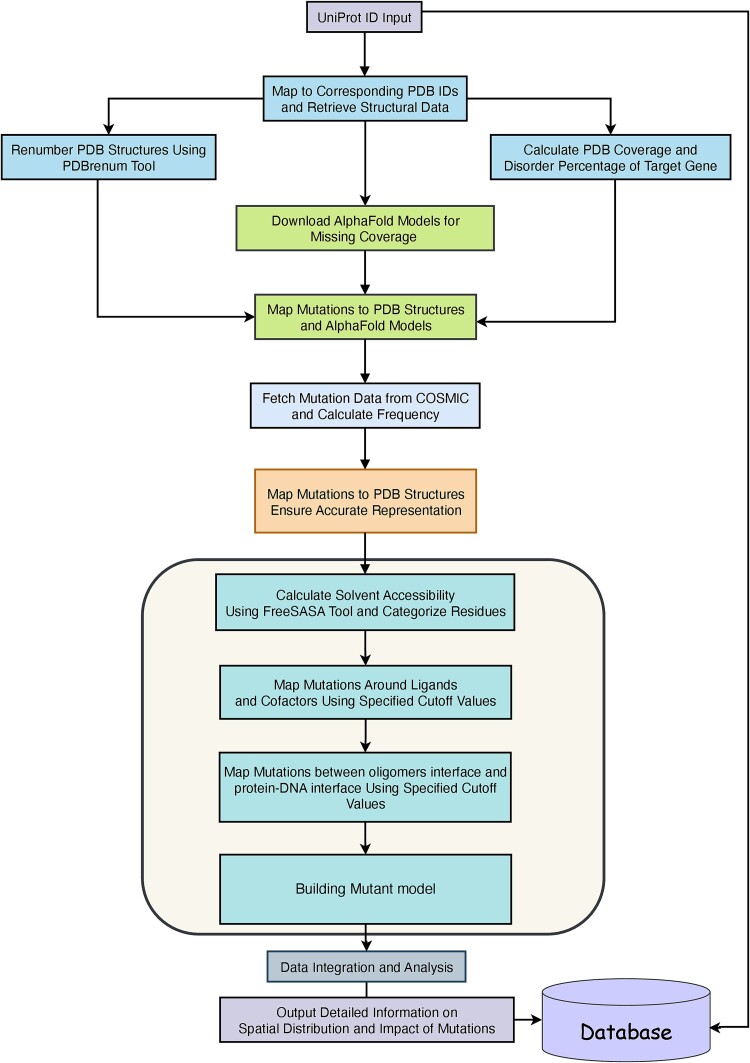
The overall workflow of the Mut-Map pipeline. The process begins with the user providing a UniProt ID or gene name. The pipeline retrieves the corresponding PDB structure from the Protein Data Bank and mutation data from the COSMIC database. These mutations are then modelled and mapped to the protein structure. The resulting data are saved to a database and subsequently displayed to the user.

Map-Mut pipeline provides a comprehensive approach to mapping and analysing mutations within protein structures, starting from a user-provided UniProt ID (Fig. 1). Initially, the pipeline maps the UniProt ID to corresponding PDB IDs to retrieve available structural data. To ensure consistent residue numbering across different data sources, the PDB structures are renumbered using the PDBrenum tool [15]. This step is very important to get the exact and correct position of the mutant on the protein 3D structure.

Next, the pipeline calculates the coverage of the target gene, identifying regions with and without structural data. Assesses the disorder percentage of the target gene to understand the extent of intrinsically disordered regions and calculates the percentage of mutations in the intrinsically disordered region. For regions lacking structural coverage or genes without PDB structures, the pipeline downloads corresponding AlphaFold models [16], ensuring comprehensive coverage by mapping mutations to both PDB structures and AlphaFold models.

Mutation data are fetched from the COSMIC database, and the frequency of each mutation is calculated to understand its prevalence. These mutations are then mapped to each PDB structure and AlphaFold model, ensuring accurate representation within the protein’s 3D context.

The FreeSASA tool is utilized to calculate the solvent accessibility of each residue [17]. Residues are categorized based on their solvent accessibility values: those with values <25 are considered core residues, those with values >80 are classified as interface residues, and those with values between 25 and 80 are considered noninterface residues. To map mutations around ligand-binding sites, a distance cutoff value of 6 Å is used, while a distance cutoff value of 5 Å is applied for mapping mutations between homo/hetero dimers and protein–DNA interface and around zinc ion cofactors.

The Modeller software [18] was utilized to generate mutant residues in the corresponding protein structures. Modeller allows for the creation of high-quality homology models by introducing specific point mutations while maintaining the overall structural integrity of the protein. This step ensures that the structural context of each mutation is preserved, enabling a more accurate analysis of how these mutations might influence protein function and stability. The generated mutant models were then integrated into the pipeline for further structural and functional analyses.

Finally, the pipeline integrates all gathered data, providing a comprehensive overview of mutation locations and their structural context. The output includes detailed information on the spatial distribution of mutations, highlighting their potential impact on protein function and interactions. This automated and integrated approach ensures a thorough analysis of mutations within the structural framework of proteins, facilitating a deeper understanding of their biological significance.

### Web server development

The web server for the Map-Mut pipeline is developed using the Python Flask framework, providing a robust and flexible backend for managing user requests and processing data. When a user submits a UniProt ID or gene name, the backend triggers the pipeline to perform the mapping and analysis tasks described earlier. The generated data are stored in multiple tables within a PostgreSQL database, ensuring efficient data management and retrieval. This database storage allows users to access previously generated data without rerunning the entire pipeline, enhancing user experience and resource efficiency.

The web server employs HTML5, Cascading Style Sheets (CSS), and Bootstrap on the front end to create a responsive and user-friendly interface. The Flask framework uses Jinja2 templating to render dynamic content in the HTML, ensuring that results and data visualizations are presented seamlessly to the user. This combination of technologies ensures that the web application is both powerful and easy to navigate, providing users with immediate access to detailed mutation mapping and structural analysis results.

## Result presentation

**Figure 2:**
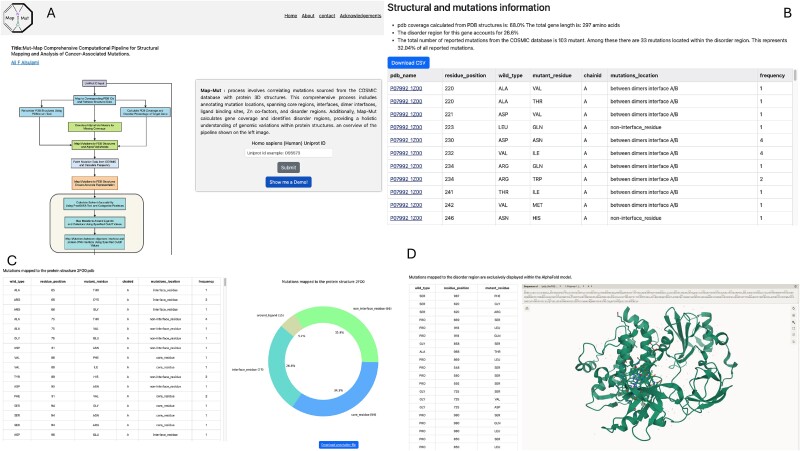
The figure illustrates the various sections of the Mut-Map pipeline website: (A) the home page provides an overview of the Mut-Map pipeline and allows users to submit their queries. (B) The results page displays a downloadable table containing comprehensive information about the target query. (C, D) The PDB page offers an in-depth analysis of the selected PDB structure, including a graph showing the location of mapped mutations, details on mutations in disordered regions, and a Mol* viewer for visualizing the selected PDB structure.

Upon completing the pipeline, the user is directed to the result page, where all data are conveniently presented in a single table (Fig. 2B). This table provides a comprehensive overview of the mapped mutations, including details such as mutation type, position, frequency, and structural context. Users can easily navigate this table to explore the mutations of interest and gain insights into their spatial distribution and potential functional consequences.

Users can select a specific structure of interest for a more in-depth analysis of mutations mapped to individual PDB structures. This selection directs them to a new page dedicated to the selected PDB, offering enhanced visualization and analysis tools. On this page, users can explore a graph depicting the percentage of mapped mutations relative to the entire protein sequence, providing valuable insights into the distribution of mutations across the structure (Fig. 2C).

In addition to the graph, users have access to a detailed table listing all mutations mapped to the selected PDB structure. This table offers comprehensive information about each mutation, including its position within the structure, mutation type, frequency, and modelled mutant. Users can easily navigate and filter this table to focus on specific mutations or regions of interest, facilitating detailed analysis and interpretation.

Furthermore, to provide users with a visual representation of the selected PDB structure, a MolStar viewer is embedded directly into the page [19] (Fig. 2D). This viewer allows users to explore the 3D structure of the protein interactively, visualize the locations of mapped mutations, and gain insights into their spatial relationships with other structural features.

Additionally, the new page includes a section for viewing mutations that appear in disordered regions of the protein, which can be visualized by selecting the corresponding AlphaFold model. This feature provides an integrated view of both ordered and disordered regions, allowing users to understand the full impact of mutations on the protein’s structure and function.

Overall, the result presentation page offers users a comprehensive and user-friendly platform for exploring and analysing mutation data mapped to protein structures. Providing both summary statistics and detailed information for individual PDB structures, along with interactive visualization tools and insights into disordered regions, the page enables users to gain valuable insights into the structural and functional implications of genomic variants.

## Discussion

The development of the Map-Mut pipeline represents a significant advancement in the field of structural bioinformatics, particularly in the context of mutation analysis. The pipeline provides a comprehensive and automated approach to mapping mutations onto protein structures by integrating multiple databases and tools [7, 8, 15, 17].

One of the key strengths of the Map-Mut pipeline is its ability to map mutations at the gene level, encompassing all available PDB structures and AlphaFold models for a given gene [20]. This comprehensive mapping ensures that mutations are analysed within all relevant structural contexts, offering a holistic view that single-structure approaches cannot provide. This gene-level perspective is crucial for understanding the full impact of mutations on protein function and interactions, as it considers all possible structural representations.

Another notable feature is the integration of mutation data from the COSMIC database. Incorporating mutation frequency data, the pipeline not only maps where mutations occur but also provides insights into their prevalence, which is essential for prioritizing mutations that may have significant biological or clinical implications. Additionally, the use of the FreeSASA tool to calculate solvent accessibility values allows for precise categorization of residues into core, interface, and noninterface regions, which is critical for understanding the potential functional consequences of mutations.

The pipeline’s ability to download and incorporate AlphaFold models addresses gaps in structural data, ensuring that even regions without available PDB structures are analysed. This feature is particularly important given the growing reliance on predictive models to fill in structural gaps. Using AlphaFold models, the Map-Mut pipeline ensures that mutations are mapped comprehensively, leveraging the latest advancements in protein structure prediction.

Moreover, the pipeline’s categorization of mutation locations, such as mapping mutations around ligand-binding sites, homo/hetero dimers, and zinc cofactors, provides a detailed spatial context. This categorization is essential for interpreting how mutations may affect protein function, stability, and interactions. For example, mutations in the core region may impact protein stability, while those at the interface might affect protein–protein interactions, and mutations near ligand-binding sites could alter binding affinity and specificity.

Integrating our pipeline with a web-based front end developed using Flask, HTML5, CSS, and Bootstrap ensures that the tool is accessible and user-friendly. Storing generated data in a PostgreSQL database allows users to retrieve previous results without rerunning the pipeline, enhancing efficiency and user experience. This web-based interface, combined with the powerful backend processing, makes the Map-Mut pipeline a valuable tool for researchers and clinicians.

The Map-Mut pipeline offers a more comprehensive and integrated solution compared to existing pipeline tools like Mutationmapper, which provides visualization of mutations on individual PDB structures [21]. However, they do not offer the same level of integration and gene-level analysis across multiple structural models. Furthermore, the COSMIC 3D platform provides valuable visualization of cancer mutations on protein structures but requires users to manually identify mutation positions, which can be time-consuming. Additionally, its PDB residue renumbering is sometimes inaccurate, potentially leading to incorrect mutation mapping. COSMIC 3D also excludes genes without experimental 3D structures, limiting its scope. In contrast, the Mut-Map pipeline automates the identification and visualization of mutation locations, ensuring accuracy through reliable renumbering. It also extends coverage to all genes by incorporating AlphaFold models, offering a more comprehensive and user-friendly solution for analysing cancer mutations at the structural level.

In addition to the current capabilities of the pipeline, future enhancements could involve the integration of multiple structural prediction tools to further enhance the analysis of mutation impacts on protein structure and function. Tools such as FoldX [22], Site Directed Mutator (SDM) [23], Maestro [23], and mutation Cutoff Scanning Matrix (mCSM) [24] offer sophisticated algorithms for predicting the stability effects of mutations based on protein structure data from the PDB. Integrating these tools into the Map-Mut pipeline enhances the accuracy and depth of mutation analysis, providing researchers and clinicians with powerful new tools for investigating the molecular basis of disease and guiding the development of targeted therapeutics.

The Map-Mut pipeline represents a valuable tool that can be seamlessly incorporated into next-generation sequencing (NGS) workflows to map variants to proteomes. As NGS technologies continue to advance, generating vast amounts of genomic data, there is a growing need for robust bioinformatics pipelines capable of interpreting this data in the context of protein structure and function. Integrating the Map-Mut pipeline into NGS workflows, researchers and clinicians can efficiently analyse genomic variants and map them to corresponding protein structures, providing crucial insights into their potential functional consequences.

### Case study

Mutations in TP53 are among the most common genetic alterations found in human cancers, occurring in over half of all tumours. These mutations often result in the loss of p53’s tumour suppressive functions, leading to uncontrolled cell growth and tumour development. Understanding the specific mutations in TP53, their locations within the protein structure, and their impact on p53’s function is crucial for several reasons, such as cancer progression, prognostic marker, and therapeutic target [25, 26].

Analysis of TP53 from Mut-Map shows the gene has 393 amino acid residues with 100% structural coverage. There are 214 PDB structures deposited for TP53 in the protein data bank. However, the entire protomer of TP53 is still not solved; each domain is solved separately with different oligomeric states. The disorder region accounts for 41% of the sequence, and 752 unique mutants are reported in COSMIC; among these, 134 unique mutations are located within the disorder region, representing 17.8% of all reported mutations.

**Figure 3:**
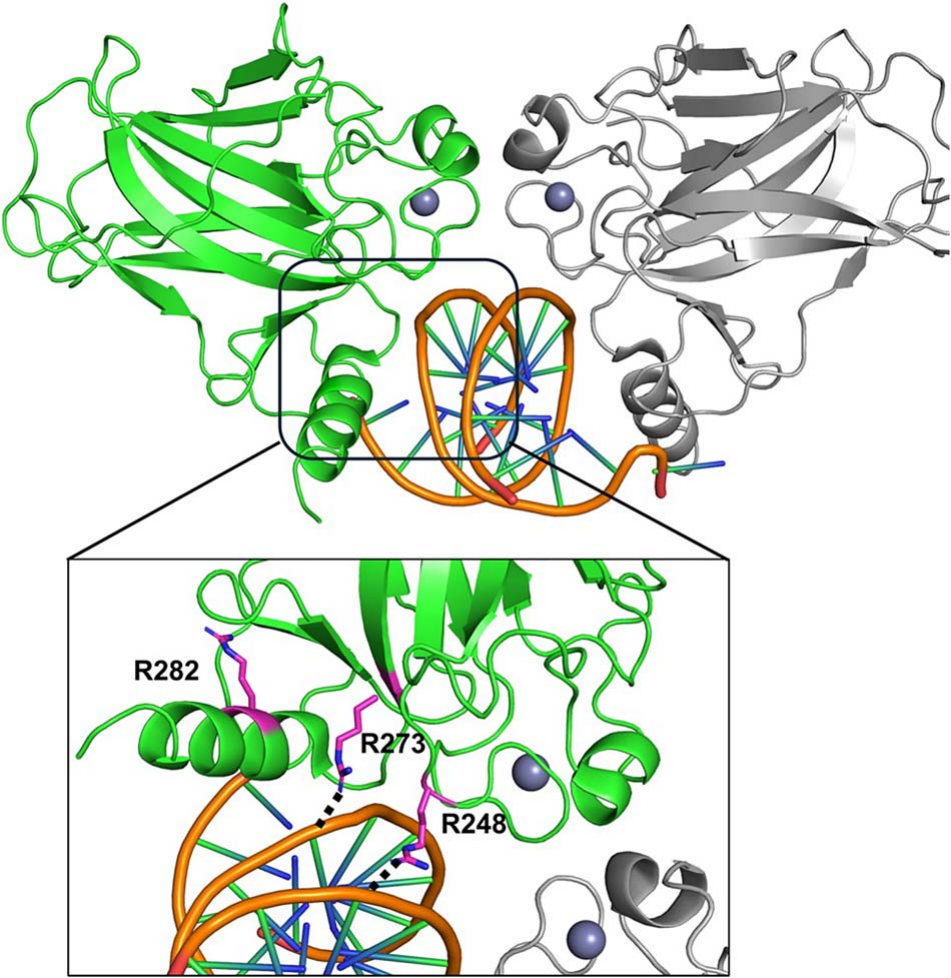
This figure illustrates a selected TP53 example from the Mut-Map pipeline. The most frequent mutant residues in the TP53 DNA-binding domain (PDB ID: 2ADY) are prominently highlighted. Hydrogen bonds between these residues and the DNA are depicted as black dots.

Looking at PDB ID:2ADY [27], 93 mutants appear at the protein DNA interface, and the most frequently reported mutations appear at the protein DNA interface are R248Q, R248W, R273H, R273C, R282W, and G245S (Fig. 3). Twelve mutations were identified around the zinc metal ion. The zinc ion is essential for the structural stability of the p53 DNA-binding domain. Mutations in this region can disrupt the coordination of the zinc ion, leading to structural destabilization and loss of function. Therefore, these hotspot mutations often lead to significant functional impairments in the p53 protein, disrupting its tumour suppressor functions.

Given TP53’s pivotal role in cancer, a comprehensive analysis of its mutations is essential for advancing our understanding of tumour biology and improving cancer treatment strategies. The Map-Mut pipeline facilitates this by mapping mutations to their structural contexts, enabling a deeper insight into the location of these mutations either between the protein–protein interface and protein–DNA interface or around the Zn cofactor.

## Conclusion

The development of the Map-Mut computational pipeline represents a significant advancement in the field of structural bioinformatics and mutation analysis. This pipeline provides a comprehensive and automated approach to mapping and analysing mutations within protein structures by integrating multiple databases and tools. Its ability to map mutations at the gene level, encompassing all available PDB structures and AlphaFold models, ensures a thorough understanding of the structural context of each mutation. The Map-Mut offers a user-friendly web interface and efficient data storage and retrieval through a PostgreSQL database; the pipeline ensures accessibility and ease of use for researchers and clinicians. This capability is crucial for rapidly translating genomic data into actionable insights for personalized medicine and therapeutic development.

The future incorporation of multiple structural prediction tools, such as FoldX, SDM, Maestro, and mCSM, will further enhance the predictive power and utility of the pipeline, providing a more nuanced understanding of how mutations affect protein stability, function, and interactions. Incorporating the pipeline into NGS workflows will enable comprehensive variant mapping and interpretation, facilitating a deeper understanding of mutation-driven disease mechanisms. Our developed pipeline is a valuable resource for the scientific and medical communities, providing the tools needed to advance research, enhance clinical diagnostics, and support the development of targeted therapies.

**Key Points**

- Comprehensive mutation mapping: The Mut-Map pipeline effectively maps somatic mutations from the COSMIC database to protein structures, providing detailed structural context and insights into mutation impacts.
- Integration of multiple data sources: The pipeline combines PDB structures, AlphaFold models, and COSMIC mutation data, ensuring comprehensive coverage and accurate analysis of protein mutations.
- Structural and functional analysis: The pipeline categorizes mutations based on solvent accessibility, DNA interface, ligand-binding sites, and dimer interfaces, offering a detailed understanding of how mutations affect protein function.
- User-friendly web interface: The Mut-Map web server, developed with the Flask framework and utilizing PostgreSQL for data storage, provides an intuitive platform for researchers to access and visualize mutation data and structural analyses.

## Supplementary Material

suplemnt_bbae514

## Data Availability

All data are freely available at: https://genemutation.org/.
