# Supplementary material for: Mut-Map: Comprehensive Computational Pipeline for Structural Mapping and Analysis of Cancer-Associated Mutations
Source: Brief Bioinform. 2024 Oct 16;25(6):bbae514. doi: 10.1093/bib/bbae514 (PMC11483132; doi:10.1093/bib/bbae514)
Supplement: suplemnt_bbae514 [file suplemnt_bbae514.docx]

Mut-Map: Comprehensive Computational Pipeline for Structural Mapping and Analysis of Cancer-Associated Mutations.

Ali F Alsulami

Corresponding author: Ali F Alsulami, Department of Biochemistry, Faculty of Science, King Abdulaziz University, Jeddah, Saudi Arabia. E-mail: afmalsulami1@kau.edu.sa

Supplementary material

Outlines the connection of the table in the database. This will help visualise the relationships between the tables.

• **main_result Table:**

- **Connected to**:
  - disorder_table through uniprot_id.
  - pdb_table through pdb_name.
- **Relationship**: One-to-many (one pdb_name can have multiple entries in main_result).

• **disorder_info Table:**

- **Connected to**:
  - disorder_table through uniprot_id.
- **Relationship**: One-to-one (one uniprot_id in disorder_info relates to one uniprot_id in disorder_table).

• **pdb_coverage Table:**

- **Connected to**:
  - disorder_info through uniprot_id.
- **Relationship**: One-to-one (one uniprot_id in pdb_coverage relates to one uniprot_id in disorder_info).

• **disorder_table Table:**

- **Connected to**:
  - main_result through uniprot_id.
  - pdb_table through uniprot_id.
- **Relationship**: One-to-many (one uniprot_id in disorder_table can relate to multiple entries in main_result).

• **pdb_table Table:**

- **Connected to**:
  - main_result through pdb_name.
  - disorder_table through uniprot_id.
- **Relationship**: One-to-many (one pdb_name in pdb_table can relate to multiple entries in main_result and disorder_table).

• **model_table Table:**

- **Connected to**:
  - pdb_table through uniprot_id.
- **Relationship**: One-to-many (one uniprot_id in model_table can relate to multiple entries in pdb_table).

• **One-to-Many Relationships**:

- main_result to pdb_table via pdb_name.
- disorder_table to main_result via uniprot_id.
- disorder_table to pdb_table via uniprot_id.
- model_table to pdb_table via uniprot_id.

• **One-to-One Relationships**:

- disorder_info to disorder_table via uniprot_id.
- pdb_coverage to disorder_info via uniprot_id.


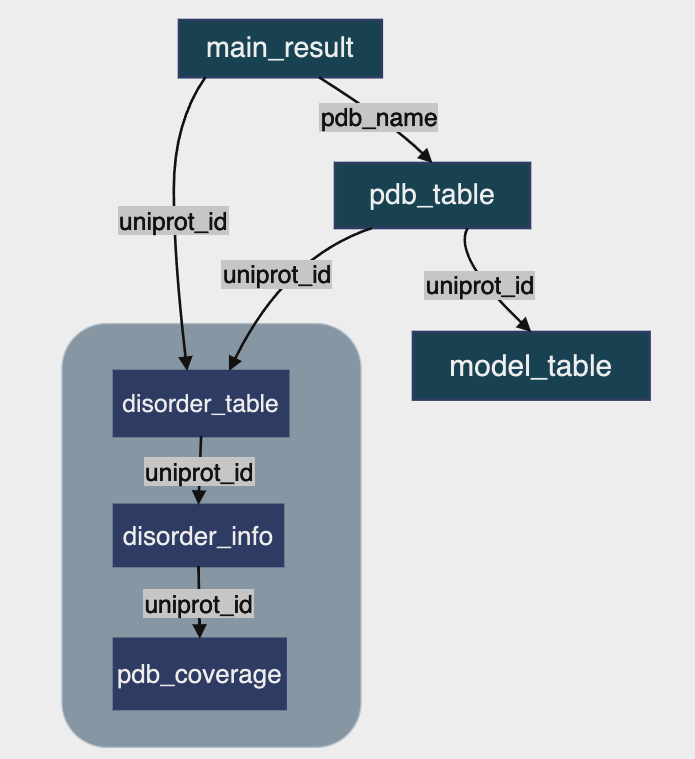


Figure S. The flowchart representing the relationships between the tables in the Map-Mut database schema.
